# Patient tilt improves efficacy of CO_2_ field-flooding in minimally invasive cardiac surgery

**DOI:** 10.1186/s13019-022-01916-5

**Published:** 2022-06-22

**Authors:** Stijn Vandenberghe, Geni Singjeli, Stefanos Demertzis

**Affiliations:** 1 Faculty of Biomedical Sciences, Università Della Svizzera Italiana, Via Giuseppe Buffi 13, 6900 Lugano, Switzerland; 2 Department of Cardiac Surgery, Istituto Cardiocentro Ticino, EOC, Lugano, Switzerland

**Keywords:** Minimally invasive, Field-flooding, Carbondioxide, Neuroprotection, Diffusor

## Abstract

**Objective:**

Space limitations during minimally invasive cardiac surgery impede consistent use of CO_2_ field-flooding. We compared different gas delivery methods, flow rates and the effect of patient inclination.

**Methods:**

A gastight model of MICS surgery with internal organs and right thoracotomy wound was created from a mannequin and equipped with a CO_2_ concentration sensor in the left ventricle. Maximum achievable CO_2_ concentration was compared for gas delivery via three commercial CO_2_ diffusors (CarbonMini, Temed, Andocor) and also via a trocar with side port. Gas flow rates of 1, 3, 5 and 8 L per minute were tested. The model was placed either in supine position or with 20° oblique tilt. A simplified transparent model was also created and placed in an optical test bench to evaluate the gas cloud motions via real-time visualization.

**Results:**

The trocar consistently achieved higher CO_2_ concentrations inside the left ventricle. At 1 l/min, approximately 2.5 min were needed to fill the supine model to its maximum CO_2_ concentration, which was limited to a range of 48–82% in the left ventricle. At higher flow rates, filling time and concentration were significantly improved. In a tilted model, all devices and all flow rates generated on average 99% CO_2_ in the ventricle. Imaging revealed constant gas exchange via the main incision, with CO_2_ outflow via bottom and air inflow via the top of the incision.

**Conclusions:**

CO_2_ field flooding in minimally invasive cardiac surgery is highly effective if the patient is tilted. Else a flow rate of 5 l/min is recommended to achieve the same protection.

**Supplementary Information:**

The online version contains supplementary material available at 10.1186/s13019-022-01916-5.

## Background

Air emboli formed during open-heart surgery result in frequent incidences of cardiac or neurological adverse events [1, 2]. On the cardiac side, transient ST elevation is fairly common but also bradycardia, right ventricular failure and reduced wall motion have been reported [3].

More recently the interest in neurological damage and cognitive dysfunction resulting from air emboli has increased thanks to improved assessment methods and imaging modalities, and the findings are astonishing. Patel and colleagues, for example, combined pre- and post-op MRI and neuropsychological tests with peri-operative quantification of air emboli via transcranial Doppler in cardiac surgery patients and counted thousands of bubbles in each patient [4]. They discovered new cerebral microbleeds in more than 80% of the patients undergoing valve surgery (significantly more than CABG) and new brain lesions and neuropsychological decline in more than 40% of the patients. Quantification of the real damage due to air embolism is difficult. Although the rate of clinically relevant stroke following cardiac procedures is relatively low [5], the high incidence of silent brain infarcts or overt lesions cannot be ignored, especially as there is evidence that these can lead to future strokes [6, 7].

Clearly, prevention is the desired approach and during open-heart surgery, this can be achieved by Carbon dioxide field-flooding, whereby blood-air contact is avoided by filling the chest with CO_2_ gas, thereby expelling all air. This technique is a simple and harmless procedure that has been safely used since 1957 during sternotomy surgery, however, its efficiency was recently questioned [8, 9]. Since the CO_2_ gas is invisible, its presence is difficult to assess during actual surgery. Therefore most information is derived via model studies, where in particular the device used for gas delivery, its positioning and the selected flow rate appear to be more important than assumed in day to day practice.

While flooding a sternotomy field with a heavier-than air gas seems as logical as filling a bowl with water, this becomes more complicated in minimally invasive cardiac surgery (MICS): space for a delivery device is limited and the incision is on the side of the thorax, thereby trapping a pocket of air at higher levels. Reduction of blood-air contact may be more focused on prevention of air entering the space and not removing air from the space once it gets in.

In this laboratory model study we tried to achieve a better understanding on how CO_2_ gas behaves during MICS surgery, while it is delivered in the patient cavity, comparing different flow rates, commercial gas delivery tools and patient’s orientation (simulating a tilted OR table). This was achieved by a realistic gastight torso model foreseen with an intraventricular CO_2_ concentration sensor [10] and by a simplified transparent thorax model where gas clouds could be visualized via the Schlieren imaging technique [9].

## Materials and methods

### MICS model

**Fig. 1:**
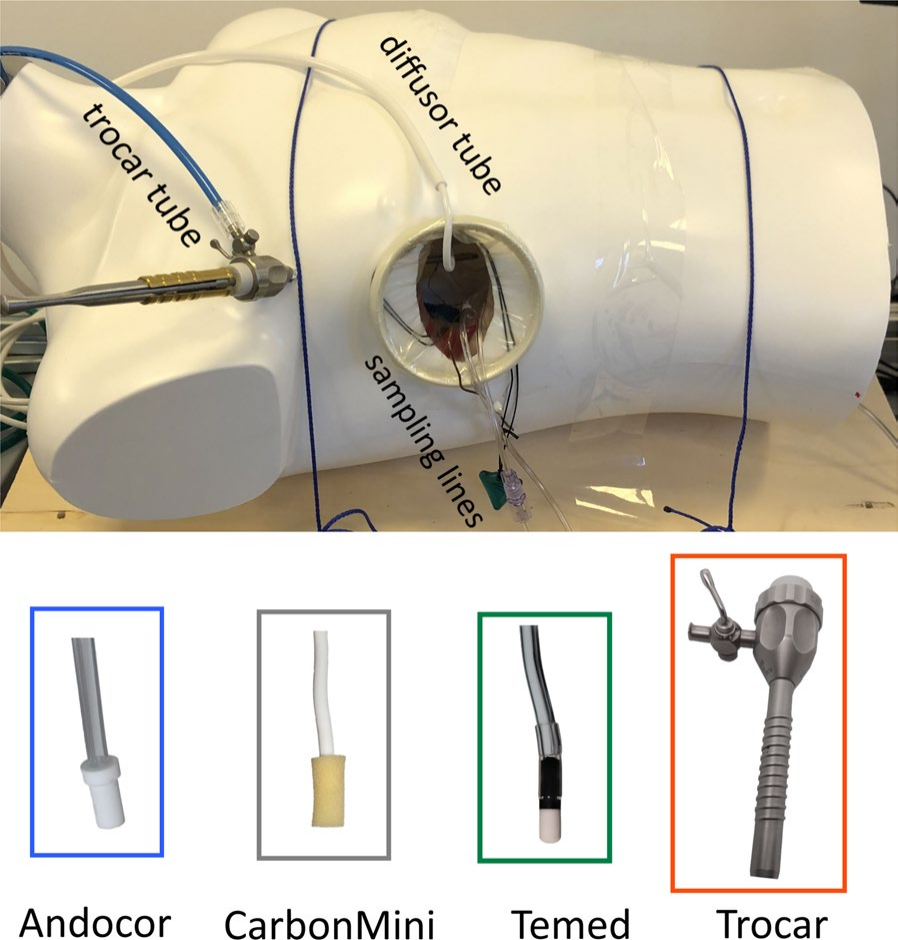
Top: the MICS model in flat position; Bottom: close-ups of the commercial CO_2_ diffuser tips and the trocar with side-port used in these experiments. For the experiments, trocar and diffuser were never inserted at the same time

The patient thorax is simulated by a hollow mannequin torso filled with a set of orthotopically organized models of various organs (heart, aorta, lungs, trachea, diaphragm, ribs, spine) embedded in a gastight mediastinum [10] with approximately 7 L of volume, including the organs. To simulate minimally invasive cardiac surgery, a hole (~ skin incision) was cut in the torso around the 5th intercostal space and the ribs were spread such that a soft retractor could be inserted (see Fig. 1). An incision was made in the left atrium of the heart model and it was spread open to provide access to the left ventricle (LV). A gas sampling line was installed through the ceiling of the LV and routed via ports in the LV wall and diaphragm outside the thorax model, while two additional sampling lines were routed via the main incision and positioned at the bottom of the model underneath the gas diffuser position (cranial) and by the diaphragm (caudal), respectively.

The model was placed on a platform consisting of two wooden plates (60 × 60 cm each) joint together with two hinges, to enable tilting and simulate the oblique inclination of the operating table at a fixed angle of 20°.

### Model for Schlieren images

**Fig. 2:**
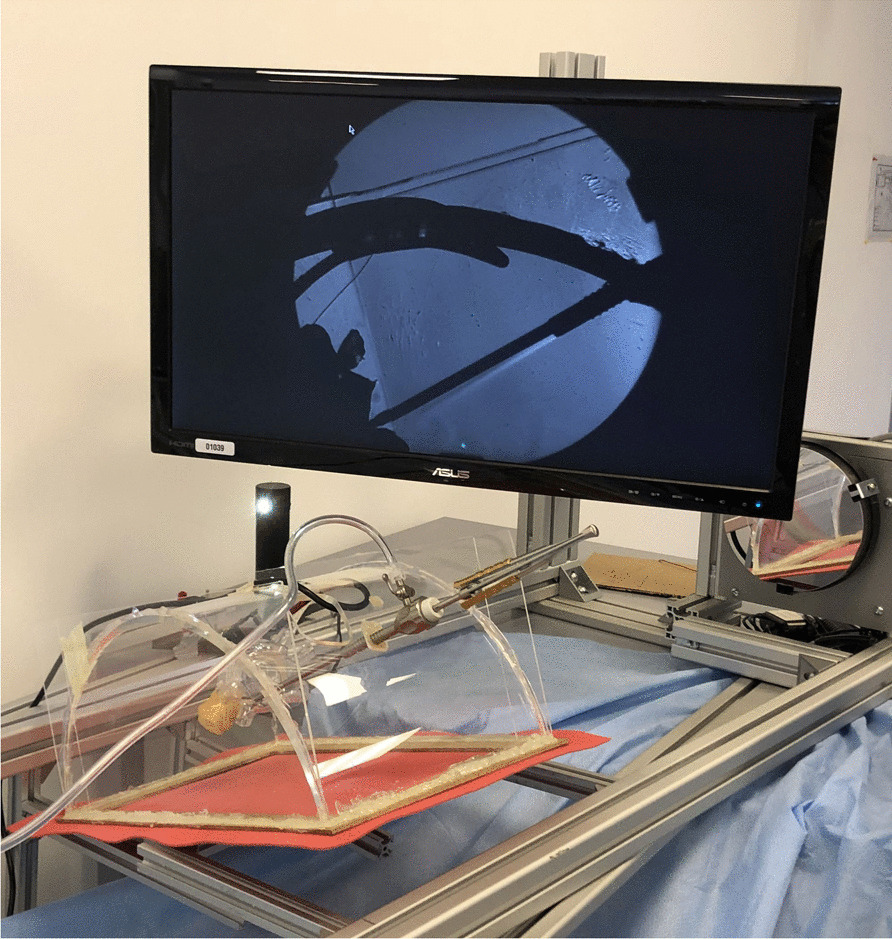
Tilted transparent thorax model in a Schlieren test bench

A Z-type Schlieren optical test bench was previously developed for visualizing CO_2_ gas clouds in a transparent sternotomy model [9]. For the same purpose, a plexiglass MICS model was created from a half-cylindrical surgery training model, which was made gas tight by welding on flat plexiglass sidewalls and a polyurethane bottom sheet (see Fig. 2). Unused trocar ports were sealed off with rubber plugs such that only the main thoracotomy window remained open. The same heart model (transparent with left atrial incision) as in the model described above was suspended orthotopically inside the thorax model on 3 strings.

The flat sidewalls of the model allow the collimated light beam of the test bench to transfer with minimal diffraction and show the Schlieren effect inside and around the thorax model. The curved surfaces of the LV model, however, strongly diffract the light beam, resulting in dark shadows blocking the view inside that LV. Details on the model and the test bench can be viewed in the supplemental video data.

### Data acquisition

The three sampling lines, composed of standard pressure line (1.5 mm ID, 150 cm length), were each connected to a membrane pump (ZR320-03PM, ZhengFuRui, Dongguan, China) and a wide-range CO_2_ concentration sensor (SprintIR—6S, Gas Sensing Solutions, Cumbernauld, UK) to monitor local CO_2_ concentrations (range 0–100%) throughout the tests. The sensors were calibrated with medical grade CO_2_ gas before each set of test runs and during the runs the three pumps generated a suction flow of 0.25 ± 0.05 l/min. In order to minimize disturbance of the gas atmosphere, this flow rate was applied intermittently for 5 s every 30 s via a timer board (XY-J02, XiongDi, China). A digital gas flowmeter with calibrations for air and CO_2_ in normalized liters/minute (MassView, Bronkhorst High-Tech BV, Ruurlo, The Netherlands) was used for control of the pump suction flow rate and recording of the delivery gas flow rate. A MATLAB script (The MathWorks Inc., Natick, MA) was used to record the digital outputs of the CO_2_ sensors and the flow meter at a sampling rate of 20 and 10 Hz, respectively.

### Gas delivery devices

Three commercially available diffusors and a trocar with sideport were tested as means of delivering CO_2_ gas for field-flooding (see Fig. 1, bottom):

- Andocor Gas Diffusor (Andocor NV, Hoogstraten, Belgium)
- CarbonMini (CardiaInnovation AB, Kungens Kurva, Sweden)
- Temed Gas Diffusor (Temed Medical Ltd., Sidlesham, UK).
- Trocar (30120EX1, Karl Storz, Tuttlingen, Germany)

The diffusors all have a similar construction in that they connect to a CO_2_ source via a ~ 3 m long, ¼” PVC tube with an inline anti-bacterial filter. This tube connects to a thinner malleable tube and ends in the actual diffusor tip. The latter part exhibits the main differences between the devices: the malleable tube of the CarbonMini has an outer diameter of ~ 4 mm and is 15 cm long, while for the Temed and Andocor devices it is ~ 6 mm with 40 cm length. The CarbonMini tip consists of a soft foam cylinder (Ø7 × 17 mm), the Temed of a hard-porous cylinder (Ø8 × 13 mm) with a dome shaped end, and the Andocor consists of a hard-porous cylinder (Ø9 × 15 mm) with a proximal flange (Ø11 × 5 mm).

These 3 diffusors, for their respective tests, were inserted at the highest point of the main thoracotomy incision with the malleable tube folded upward so the tip would sit against the internal anterior thoracic wall. This position is typically used clinically and places the diffusors horizontally, in contrast with usage in a sternotomy procedure where they should, according to all ‘Instructions For Use’, be bent downwards.

The fourth gas delivery system, the threaded metal trocar with a lateral stopcock, was screwed through the plastic wall of the mannequin and also penetrated the gastight mediastinum compartment via a rubber seal. The stopcock was used to connect the CO_2_ supply such that the gap between the trocar inner lumen and the inserted instrument shaft acted as a tunnel for CO_2_ delivery inside the thorax.

### Data collection protocol

Data was recorded for all combinations of the following factors:

- 4 different gas delivery systems
- 4 different CO_2_ flows (1, 3, 5 and 8 l/min)
- 2 tilting angles of the mannequin (0° and 20°)

for a total of 32 tests, executed sequentially in one run. Five runs (repetitions) with the 32 tests were performed, whereby the order of tests was randomized within each run. Each test starts by purging the model of CO_2_ by removing the lid representing the diaphragm and using a fan inside until all concentration sensors indicated < 1% CO_2_. Next, the selected delivery tool was installed and the tilt angle of the model set according to the selected test. The timer boards and sampling pumps were then activated and finally the MATLAB script for recording was started simultaneously with the start of CO_2_ delivery flow. The recording stopped automatically after 800s, after which all active components were manually turned off and the model was purged again.

A similar protocol was used for the gas cloud visualization in the transparent thorax model, with the exception that no repetitions were performed and imaging was not performed at 8 l/min. The CO_2_ gas in this model was removed with a laboratory vacuum pump in between recordings.

### Data analysis

All concentration data acquired in these tests follows a similar hyperbolic curve pattern: starting at 0%, a steep rise in concentration occurs and then tapers off, leading to a steady plateau which is maintained until the end of the recording [9]. Therefore, after filtering of the data (IIR low-pass filter of order 50 with 50 mHz cut-off frequency), two parameters were extracted: the maximum CO_2_ concentration achieved (i.e., the average of the final plateau), and the rise time (i.e., time needed to reach 90% of the plateau).

This exploratory study is based on the findings in one virtual patient, and is intended to guide parameter (factors and levels) selection for future clinical studies. In model data collected in a controlled environment, the variation is expected to be much smaller than in a clinical setting and for this and the limited repetitions, the statistical analysis of the model data is limited to non-parametric multi-level comparisons, where Kruskal–Wallis rank sum test with pairwise Dunn correction was used for evaluating the effect of a single factor and Friedmann’s test was used for two-factor comparisons. All tests were performed with MATLAB software and a value of p < 0.05 was considered significant.

## Results

### Overall evaluation

**Fig. 3:**
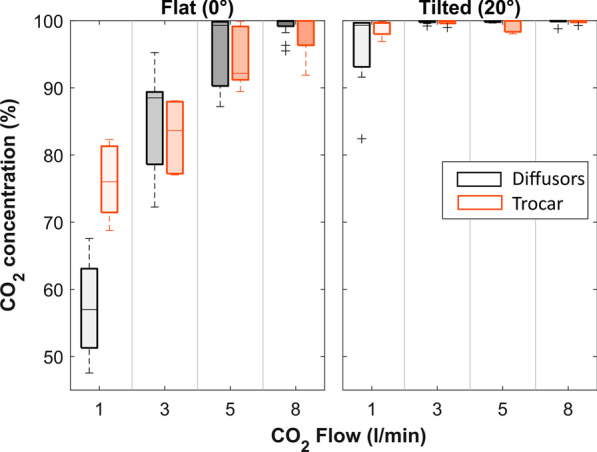
Boxplots indicating the max CO_2_ concentrations that were measured inside the Left Ventricle for the different conditions, whereby the gray boxes represent the pooled data of the three commercial diffusors and the orange boxes the data obtained with the trocar

**Fig. 4:**
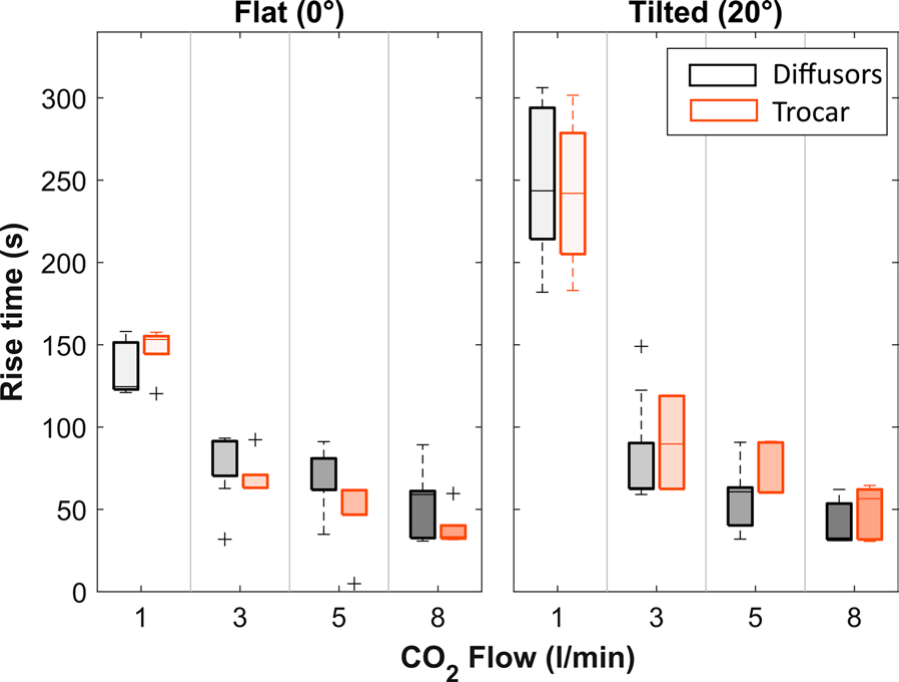
Boxplots indicating the Rise time to achieve maximum CO_2_ concentration inside the Left Ventricle for different conditions. Gray boxes: commercial diffusors (pooled); Orange boxes: trocar as gas delivery device

For the purpose of simplicity, only the data recorded inside the left ventricle (LV) is presented below (Fig. 3, Fig. 4 and Additional file 1) as this is the most critical parameter to assess the embolic risk for the patient.

In general, the 3 factors evaluated (gas delivery device, flow rate, and patient tilt) influence both the maximum achievable CO_2_ concentration in the thorax and within the left ventricle and the time to achieve this concentration. The effect of each factor is discussed in detail below. Overall, the rise time inside the LV ranged from 31 s (at 8 l/min) to more than 5 min (306 s at 1 l/min), while the CO_2_ concentration reached inside the LV ranged from 47% (flat model at 1 l/min) to 100% (majority of the test combinations).

The rise time of the concentration inside the left ventricle is highly dependent on the delivery flow rate, and slight, non-significant, differences were observed between the diffusers (Fig. 4). Also the effect of tilting plays a role, where the orientation of the LV with respect to the location of the diffuser and gravity are in a different constellation.

Hence, when the model is positioned flat, the trocar with 8 l/min CO_2_ output reaches a plateau the fastest (38 ± 11 s), while Andocor and Temed diffusers both need 49 ± 14 s. In a tilted model, the situation is exactly reversed with the trocar needing 49 ± 15 s at 8 l/min while the Temed and Andocor diffusers then fill the LV the fastest and reach a plateau in 38 ± 11 s.

### Effect of gas delivery device

Despite slight differences, the commercial diffusers that are all placed in the same position in the model yield similar results over the whole range of test conditions. At 1 and 8 l/min in the flat position, for example, the following concentrations were respectively achieved: Andocor 59/99%; CarbonMini 55/100%; Temed 57/99%. Friedman analysis with adjustment for the flow rates indicated no significant difference with pairwise, Dunn-corrected p-values all > 0.831 and therefore the data of the commercial diffusers was pooled together in Figs. 3 and 4 and for further analyses.

A significantly better performance in maximum CO_2_ concentration was achieved with the trocar at 1 l/min when compared to the commercial diffusers in a supine model (*p* = 0.001), but this difference is no longer significant for other flow rates or when the model is inclined. Related, it appears that in a supine model the trocar gas delivery is quite robust and less dependent on flow rate, while the commercial diffusers demonstrate a high sensitivity, especially at low gas flow rates, where an increase in flow from 1 to 3 l/min results in a 28% jump in CO_2_ concentration, to reach 85% on average. In the tilted model, the measured maximum concentrations are much closer to full saturation, with an IQR of [99.6–99.9%] and therefore device selection is insignificant.

### Effect of flow rate

An increase in the CO_2_ delivery flow rate increases the maximum achievable CO_2_ concentration, and also reduces the time needed to achieve this concentration (Fig. 4). This confirms that in order to get a complete filling of the thorax, a volume of CO_2_ has to be delivered that is many times higher than the actual internal volume of the thorax cavity. The effect of flow rate on rise time and CO_2_ concentration is most obvious when considering 1 l/min versus 3 l/min delivery flow. For the commercial diffusers, a highly significant improvement is achieved for concentration (p < 0.001) in a flat model only, while a significant reduction in rise time is achieved in the flat (p = 0.005) and the tilted model (p < 0.003). When even higher flow rates are considered, there is still a positive impact of further increasing the flow, but it is no longer significant. In the flat model, however, a flow rate of 5 l/min ‘only’ leads to an average concentration of 95% CO_2_ (range 87–100%) for all devices combined, which arguably is not ‘full embolic protection’. An additional effect of increased flow rate, for both rise time and concentration, is that the variability between repetitions is reduced, indicating a higher level of stability and that the actual level of protection will be less dependent on uncontrolled factors.

### Effect of tilting

Tilting the model eliminates the effects of flow rate and device on the maximum achievable concentration, with an overall median of 99.8% CO_2_ in the LV and also greatly reduces variability [IQR: 99.6–99.9%]. The exception is 1 l/min, with its larger spread and 1st quartile at 94%, its performance still differs significantly (p < 0.002) from the concentrations achieved at all the other flow rates. Still, the biggest impact of simply tilting is seen at this 1 l/min gas flow rate, where the concentration jumps from an average of 57–96% for the commercial diffusers and from 76 to 99% for the trocar.

The tilting angle is not a key factor for the rising time. Where tilting always results in an improvement in the concentration, it effectively extends the rise time and thus requires more time to sufficiently fill the thorax with CO_2_ gas. Again, a highly significant impact is seen at 1 l/min, where the rising time jumps from a mean of 138–245 s when the model is titled.

### Gas visualization

**Fig. 5:**
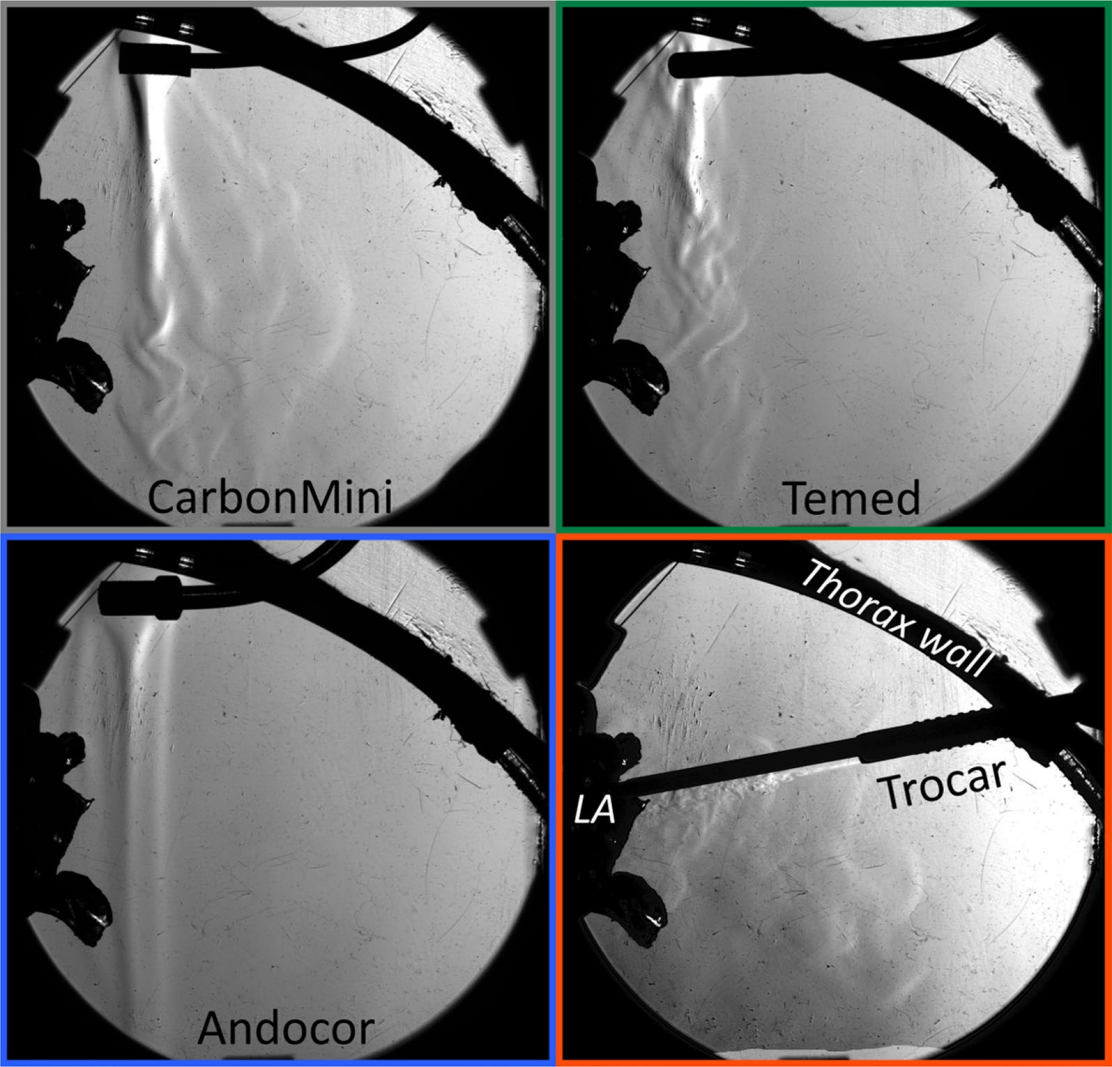
Snapshots from the Schlieren imaging for the different diffusers in a supine model at 3 l/min. Video of the gas clouds at different flow rates is presented in Additional files 2 and 3

The Schlieren setup succeeded in contrasting CO_2_ gas clouds with the surrounding air (see video segments in Additional files 2 and 3). Gas flows from commercial diffusors installed against the anterior wall show that almost all flows are gently delivered and the gas falls down with gravity (Fig. 5), apparently directly in the left atrium in the tilted model. This latter observation may be deceiving as it concerns a projection and the gas may also fall in front of or after the organ and would yield the same imaging. Only the Temed diffuser generated some jets from the proximal part of the tip when the flow was set at 5 l/min.

A jet is basically the working principle of the trocar where the gas is forced through the radial gap between the trocar and the instrument and is thereby guided directly towards the opened left atrium. The video with the tilted model at 5 l/min also provides a glimpse of the CO_2_ flow through a trocar without an instrument and in general, the 3 and 5 l/min flow rates demonstrate a high degree of turbulences in the jet. Careful observation shows that the gas is propelled into the heart and bounces back or overflows from the bottom of the atrial incision.

## Discussion

Carbon dioxide field-flooding during a sternotomy or thoracotomy procedure is based on the same principle: the thorax is filled from bottom up until it overflows and pushes away all the air that can surround or enter the heart. The main difference related to field-flooding effectiveness is that a sternotomy has the incision at the highest point of the thorax, which acts as a bowl, while a thoracotomy patient resembles more a covered bucket with a hole in the side. As such, the dense gas flows out of the lower part of the incision while air flows in from the highest part of the incision to replace the lost volume. This is not only an assumption but was clearly observed in the Schlieren experiments, where gas was seen exiting the incision and streaming down along the model’s side.

This in vitro study was performed to answer some practical questions related to CO_2_ field-flooding in MICS surgery and as a primer for future clinical studies. The two self-evident questions are: “does it matter which device is used to deliver CO_2_ gas?” and “which is the lowest flow rate necessary to get good field-flooding?”. However, a third factor was included because during MICS surgery the operating table occasionally gets tilted to provide a better view for the surgeon and a better orientation of the organs and since field-flooding is gravity based, an effect of tilting was hypothesized.

Our data shows that tilting is in fact the most important factor that guarantees a safe environment to avoid blood-air contact. We further demonstrated that commercial diffusers are not yielding any different results, but that using the side port of a trocar is in fact an acceptable or even superior method for delivering the gas in the thorax.

As known from sternotomy model studies, the higher the flow rate, the faster the surgery field gets flooded and the higher the concentration in the thorax can get [9, 11]. This is clearly a dynamic process where the gas delivery needs to continue because otherwise diffusion and convection rapidly increase the air content inside the thorax [9, 10, 12]. The same statements are valid for this MICS model, where a flow rate of only 1 l/min has some specific concerns that fade at higher flows. Namely only at 1 l/min is there a significantly lower concentration and higher rise time than at the other flow rates, and only at 1 l/min do commercial diffusers perform significantly worse than a trocar as gas delivery device.

Currently, robust clinical evaluation methods for the effects of air embolization are non-existent and hence it is impossible to set a hard cutoff to distinguish between sufficient and insufficient or safe and unsafe concentrations of CO_2_ in the surgical field. If we assume that a 90% CO_2_ concentration could drastically improve patient outcomes, then our data suggests that in a supine patient a flow rate of at least 5 l/min should be applied, while in a tilted patient any flow rate would do. However, this does not take into account that during a surgery the CO_2_ atmosphere is constantly disturbed by motions and vents, and intermittently by a blood sucker whose flow rate can be as high as 25 l/min and thus eliminate all CO_2_ within seconds. This is where the consideration of rise time becomes important: a short rise time can supposedly quickly rebuild a CO_2_ atmosphere and provide some robustness against constant disturbances. The rise times observed here are surprisingly long and thus, even in a tilted patient, the authors’ recommendation would be a minimal CO_2_ flow rate of 5 l/min, where it can still take a full minute to rebuild a safe atmosphere after the application of strong suction.

One has to take into account that our rise time measurement is based on a worst case scenario where a measurement is started with an opened thorax completely deprived of CO_2_, while in reality many surgeons already start the insufflation as soon as the first small incision is made, thereby attempting to prevent air from entering in the first place. Furthermore, a limitation of our rise time interpretation is that it is based on intermittent sampling, rather than continuous, to minimize corruption of the CO_2_ atmosphere. Lastly, the rise time data should be interpreted in parallel with the concentration data because it is the time to reach the maximum concentration for a specific condition, which is not always 100%. Considering this puts the results of 1 l/min gas delivery in an extra negative perspective: not only are several minutes needed to reach a steady CO_2_ atmosphere, that atmosphere provides very weak protection with a CO_2_ concentration of only 57%.

**Fig. 6:**
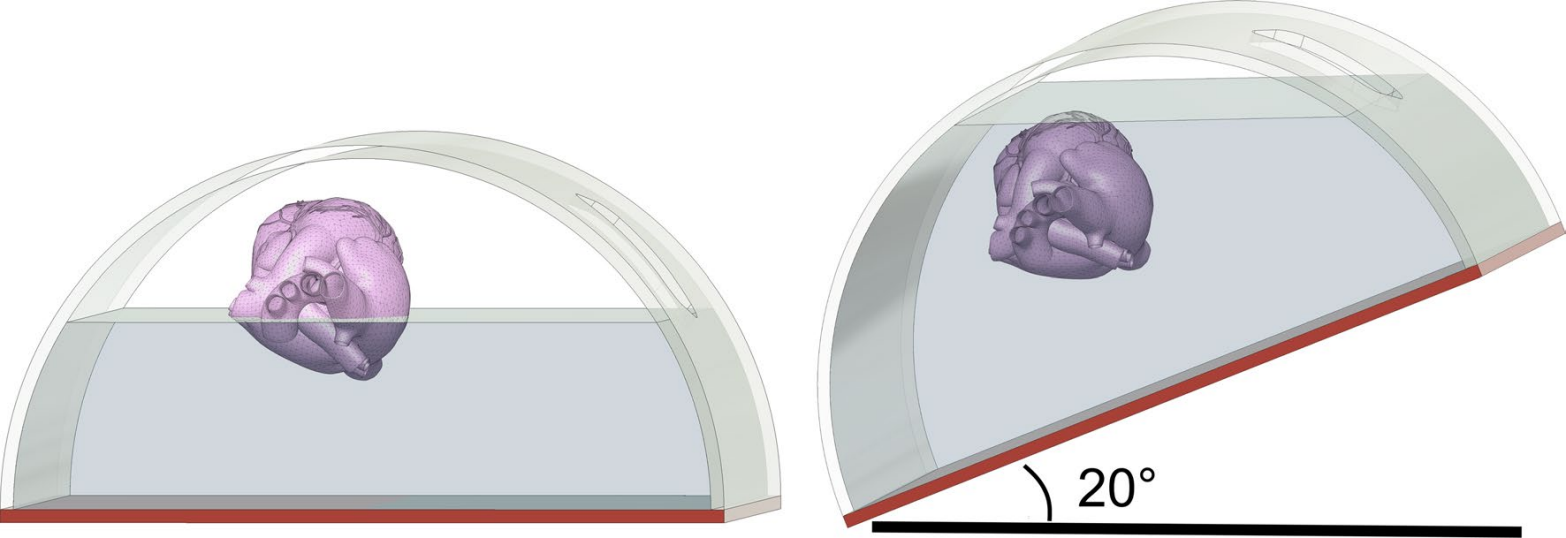
Hypothesis on the effect of patient tilt illustrated with the Schlieren model: the heart gets better submerged in CO_2_ because the incision comes higher than the heart. Conversely, more CO_2_ can be contained in the thorax and the filling time to submerge the heart will be longer

In order to explain the negative effect of tilting on rise time and the superior positive effect on maximum concentration, Fig. 6 depict the supine and tilted thorax as seen in our Schlieren experiments. In a supine patient, the heart can never be completely submerged in CO_2_ gas because it spills from the lower end of the incision, unless delivery flow rates exceed the drainage rate. When tilted, however, the point of spillage is raised and thus the heart can be submerged, but this is paired with a larger volume that needs to be filled before the heart is reached, and thus a longer rise time.

Even though a model study is currently the best option for obtaining detailed concentration data within a thorax under controlled circumstances, it is always a simplification of reality and bound to limitations. In this case we were able to place the commercial diffusers for every test in exactly the same position in the thorax based on visible markers (the mannequin is slightly translucent), while in reality there will be far more variation in the diffusor placement. Especially if a more cranial or caudal position is chosen and the CO_2_ does not fall directly on/in the heart, lower concentrations can be expected, especially in the supine position. Clinically, there will also be greater variation caused by the diverse patient anatomies and our results may differ from reality due to our simplified anatomic representation. This simplification also did not allow us to evaluate what happens with air that is trapped in the upper pulmonary veins or in other pockets. Can CO_2_ insufflation remove this air via convection or diffusion, or will it stay trapped until it embolizes or is eliminated by a de-airing manoeuvre?

## Conclusion

The use of carbon dioxide gas for field-flooding also works in minimally invasive cardiac surgery to remove intra-thoracic air. Besides commercial gas diffusers, which all yield similar results, a trocar with side port can be considered as an effective alternative for gas delivery. This model study shows that a delivery flow rate of at least 3 l/min is indicated, but the most beneficial action to increase the effectiveness of field-flooding is to tilt the patient.

## Supplementary Information

**Additional file 1.** Spreadhseet summary of all concentration and rise time data by the Matlab script.

**Additional file 2.** Video presentation of the Schlieren gas visualization results in a supine MICS model.

**Additional file 3.** Video presentation of the Schlieren gas visualization results in a 20° tilted MICS model.

## Data Availability

The dataset containing the concentration and rise time data extracted from all the recorded runs and supporting the conclusions of this article is included within the the additional file “Additional file 1.xlsx”. A summary of the optical test bench results displaying the gas clouds for all diffusers at flow rates 1, 3, and 5 l/min is included in the additional files “Additional file 2—Schlieren—Flat.mp4” and “Additional file 3—Schlieren—tilted.mp4”.

## Abbreviations

MICS: Minimally invasive cardiac surgery
CO_2_: Carbon dioxide (gas)
PMMA: Polymethylmethacrylate
SLPM: Standard liter per minute (gas volumetric flow rate scaled to standard temperature and pressure (0 °C, 1 bar)). In the text, all indications of l/min are in SLPM
LV: Left ventricle
LA: Left atrium
